# Low-grade extraskeletal osteosarcoma of the chest wall: case report and review of literature

**DOI:** 10.1186/1471-2407-10-645

**Published:** 2010-11-24

**Authors:** Renaud Sabatier, Corinne Bouvier, Gonzague de Pinieux, Anthony Sarran, Isabelle Brenot-Rossi, Florence Pedeutour, Bruno Chetaille, Patrice Viens, Pierre-Jean Weiller, François Bertucci

**Affiliations:** 1 Department of Medical Oncology, Institut Paoli-Calmettes, 13009 Marseille, France; 2 Department of Pathology, CHU La Timone, 13005 Marseille, France; 3 Department of Pathology, Hôpital Trousseau, 37000 Tours, France; 4 Department of Radiology, Institut Paoli-Calmettes, 13009 Marseille, France; 5 Department of Nuclear Medicine, Institut Paoli-Calmettes, 13009 Marseille, France; 6 Laboratory of Solid Tumours Genetics, University of Nice-Sophia-Antipolis, CNRS UMR 6543, Nice University Hospital, 06000 Nice, France; 7 Department of Pathology, Institut Paoli-Calmettes, 13009 Marseille, France; 8 Department of Internal Medicine, CHU La Timone, 13005 Marseille, France; 9 University of Mediterranea, Marseille, France

## Abstract

**Background:**

Low-grade extraskeletal osteosarcomas (ESOS) are extremely rare.

**Case presentation:**

We present the first case of low-grade ESOS of the chest wall, which occurred in a 30-year-old man. Because of initial misdiagnosis and patient's refusal of surgery, the diagnosis was done after a 4-year history of a slowly growing mass in soft tissues, leading to a huge (30-cm diameter) calcified mass locally extended over the left chest wall. Final diagnosis was helped by molecular analysis of *MDM2 *and *CDK4 *oncogenes. Unfortunately, at this time, no surgical treatment was possible due to loco-regional extension, and despite chemotherapy, the patient died one year after diagnosis, five years after the first symptoms.

**Conclusion:**

We describe the clinical, radiological and bio-pathological features of this unique case, and review the literature concerning low-grade ESOS. Our case highlights the diagnostic difficulties for such very rare tumours and the interest of molecular analysis in ambiguous cases.

## Background

Osteosarcoma (OS) typically develops in the intramedullary cavity of long bones of adolescents and young adults and is a high-grade tumour [1,2]. Extraskeletal osteosarcoma (ESOS) is a very rare form of OS (~5%) located in the soft tissues without connection to the skeleton. Its usual aspect is that of a large and deep high-grade bone-forming sarcoma, developed in the limbs of patients older than 40 years [2,3], with a very aggressive behaviour [4].

Low-grade ESOS, whose histology is similar to well-differentiated intraosseous and parosteal OS, is extremely rare. To date only six cases have been reported in the literature. Here, we describe a new case of low-grade ESOS, which developed in the chest wall. Initial diagnosis, based on radiological and histological aspects, was erroneously myositis ossificans. Correct final diagnosis, suspected by the clinical evolution, was confirmed by molecular analysis, but was too late, and the patient died from extensive loco-regional progression. We hope that this case report underlines the diagnostic difficulties of this tumour, and the interest of molecular analysis in ambiguous cases.

## Case presentation

The patient was a 30-year-old man, Caucasian type, without any specific medical personal or familial history. He was referred to our institution in November 2006 for diagnosis and treatment of a huge tumour of the left chest wall, detected for the first time 4 years earlier. Indeed, in May 2002, he presented a 3-cm painless, mobile, intramuscular mass on the left shoulder near scapula, three months after a benign trauma on this region. Magnetic resonance imaging (MRI) showed elements in favour of a myositis ossificans circumscripta. Percutaneous biopsy showed areas of osteogenesis with mature bone trabeculae in muscle. The intertrabecular space revealed benign-appearing fibroblastic proliferation. The retained diagnosis was myositis ossificans. The benignity of this disease with usual spontaneous stabilisation or regression [5], and the lack of functional impact of the lesion led the physicians to decide observation.

**Figure 1:**
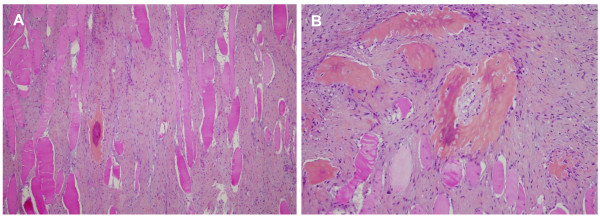
**Microscopic features**. (**A**) Proliferation of fusiform cells between muscle fibres (HES × 10). (**B**) Foci of osteogenesis with bland fusiform cells and no mitosis (HES × 20).

However, the patient was lost to follow-up, and the mass continued to grow slowly during two years. A second percutaneous biopsy was performed in May 2004. Histological analysis showed an intramuscular tumour made of bland fusiform cells with few mitoses and mature bone trabeculae (Figure 1). The morphological features were quite similar to that of the first biopsy, but with some worrisome features such as the absence of classical "zonal phenomenon", and more florid fusiform proliferation and bone forming. Because of the long clinical history and the pathological aspect, the diagnoses of "atypical" myositis ossificans or heterotopic ossification were evoked and a surgical removal was proposed. The patient refused, and consulted another physician who prescribed diphosphonate treatment during a few months.

**Figure 2:**
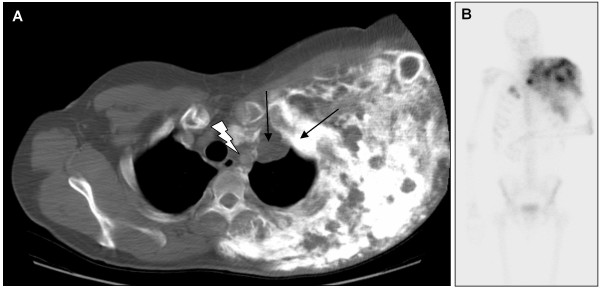
**Radiological features 4.5 years after the first symptoms**. (**A**) CT scan: thoracic transversal view showing a voluminous heterogeneous highly calcified mass. It included the whole left chest wall and the left scapula, with intercostal invasion to the apical pleura (black arrows) and the antero-superior mediastinum (bolt) with trachea deflection to the right. (**B**) Bone scan with Technecium 99: see the heterogeneous extended hyperfixation of the whole left side of the chest. There was a muscular extension to the triceps leading to a frozen scapulo-humeral joint.

The patient was then lost to follow-up during 17 months until November 2006, when he was referred to our institution because the mass had kept growing, leading to a shoulder immobilisation. The ECOG status was 1, and the patient presented a frozen left shoulder associated with ipsilateral radial and cubital paralysis and cervical pain. Physical examination found a voluminous, plane, hard and immobile mass extended from the basicervical region to the scapulo-humeral joint and from the left side of the chest wall to the axillary region. Biologically, there was not any abnormality except an elevation of serum alkaline phosphatases. Radiological images were impressive. CT scan showed a huge calcified heterogeneous mass, with kystic and solid elements (Figure 2A). The mass had a 25-cm transversal diameter, an 18-cm antero-posterior diameter and a 30-cm cranio-caudal diameter. It included the left scapula, the periscapular muscles and the chest wall, with intercostal invasion to the pleura and muscular extension to the left arm (triceps). Tc99 bone scan showed a large hyperfixation of the whole left side of the chest (Figure 2B). PET-scan with 18-FDG showed the mass localised in the chest left side, crossing the median line at the first sternocostal joint. Hyperfixation was heterogeneous and moderate with a few highly metabolic zones. A subcutaneous lesion was also observed in the right pectoral muscle (SUV max = 7 g/ml), as well as pleural abnormalities.

**Figure 3:**
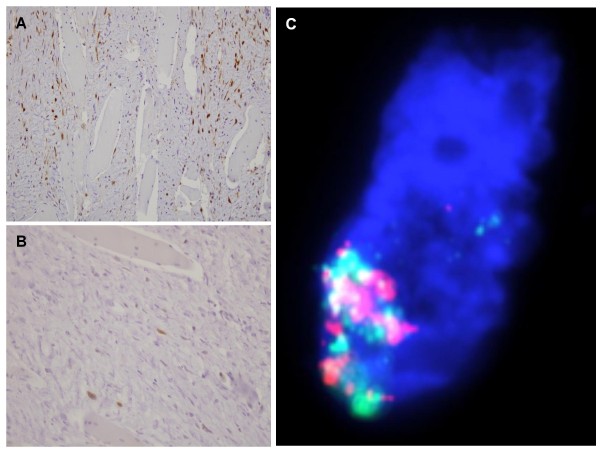
**CDK4 and MDM2 analysis**. (**A**) Immunohistochemistry with anti-CDK4 antibody showing strong and quite diffuse nuclear staining of fusiform cells (× 20). (**B**) Immunohistochemistry with anti-MDM2 antibody showing a more focal nuclear positivity. (**C**) Fluorescence *in situ *hybridization of one neoplasic cell: high nuclear amplification of *CDK4 *(red signals) and *MDM2 *(green signals), with more than 20 copies (× 40).

The diagnosis of low-grade ESOS was strongly suspected. For confirmation, the pathological samples removed in May 2004 were collected for re-examination, and notably for molecular analysis of *MDM2 *and *CDK4 *oncogenes, whose amplification had been reported in low-grade intraosseous and parosteal osteosarcomas [6-9]. As shown in Figure 3A-B, CDK4 displayed a strong nuclear staining of fusiform cells by immunohistochemistry (IHC), whereas MDM2 had a more focal positivity. Fluorescence *in situ *hybridization (FISH) analysis showed high amplification for both *MDM2 *and *CDK4 *(Figure 3C), confirming the diagnosis of low-grade ESOS.

Unfortunately at this time, more than 4 years after the first symptoms, curative surgical resection was not possible because of to the loco-regional extension. We delivered chemotherapy with an API/AI sequential regimen, which combined adriamycin (A), cisplatinum (P) and ifosfamide (I). The disease was stable after 2 API/AI cycles. After 4 additional API cycles, the disease progressed with extension of cutaneous nodules and tumour mass associated to the apparition of left pleuritis. The ECOG status was 3, and the patient complained of thoracic pain and dyspnoea. Palliative radiation therapy was not possible technically due to the tumour volume. A thoracoscopic talcage was done, followed by trabectedin-based chemotherapy. Three weeks after the first cycle, the patient deteriorated rapidly and died of respiratory failure secondary to loco-regional extension, 5 years after the initial symptoms.

## Discussion

Low-grade ESOS are extremely rare with only 6 cases reported to date in the English literature since 1953 [10-14]. To our knowledge, the present case is the seventh case reported, and the first one developed in the chest wall.

These cases are summarised in Table 1. The sex ratio is 4 F/3 M, and the median age is 40 years. These tumours likely arise from the sarcomatous transformation of multipotent mesenchymal cells contained in soft tissues. Evolution is slow, and the diagnosis is delayed with a median interval after the discovery of the mass of 4 years (range, 2 to 10 years). This delay explains the large size of tumours at diagnosis (median 14 cm, ranging from 5 to 30 cm). However, high-grade histological features (dedifferentiation and high mitotic activity) may appear after a long period in analogy to what happens in low-grade parosteal OS, thus leading to accelerated evolution, like in our case with rapid loco-regional extension, or in two cases with metastatic diffusion [10,14]. Early diagnosis of this tumour is crucial to allow an adequate surgical resection, which represents the sole curative treatment, as confirmed by the prolonged complete remissions observed in the 4 out of 5 patients initially treated with complete surgery.

**Table 1:** Seven cases of low-grade ESOS reported in literature.

Ref.	Sex/Age	Initial diagnosis	Tumour location	Largest diameter	Treatment	Clinical outcome
[10]	M/44	Myositis ossificans	Right thigh	5 cm	S	Lung metastases, 4 years, death
[11]	F/57	Low-grade ESOS	Right popliteal fossa	24 cm	S	Disease-free, 5 years
[12]	F/74	Parosteal OS	Left axilla	14 cm	S	Disease-free, 2 years
[13]	F/35	Myositis ossificans	Left leg	11 cm	S	Disease-free, 4 years
[14]	F/40	Parosteal OS	Back, para-spinal	9 cm	S	Lost to follow-up, 2 months
[14]	M/32	Low-grade ESOS	Right thigh and others	16 cm	S + CT	Lung and retroperitoneum metastases, 4 years, death
Our case	M/30	Myositis ossificans	Chest	30 cm	CT	Loco-regional extension, death 5 years after first symptoms and 1 year after diagnosis

M, male; F, female; ESOS, extraskeletal osteosarcoma; OS, osteosarcoma; S, surgery; CT, chemotherapy

However, differential diagnosis may be problematic at early stages with other ossified lesions of soft tissues: benign lesions such as myositis ossificans circumscripta (MOC), ossifying lipoma, soft tissue osteoma or chondroma, and ossifying fibromyxoid tumour, as well as malignant lesions such as classical high-grade ESOS, parosteal OS, mesenchymal chondrosarcoma, and synovial sarcoma. MOC is a benign heterotopic ossification of soft tissues characteristically associated with direct trauma [15]. It is one of the most important differential diagnoses, which was initially evoked - based on radiological and/or pathological aspects - in at least 3 out of the 7 reported cases of low-grade ESOS. Furthermore, a number of cases of ESOS presumably arising from MOC have been reported [16-19]. In MOC, both mature and immature bone is seen, as well as a prominent spindle cell and chondroid component, with a specific architecture described as the "zonal phenomenon". This phenomenon refers to the presence of a clearly benign, reactive rim of mature bone at the periphery, which encompasses a central area of fusiform cells and immature bone interlaced with osteoid and chondroid. In our patient, several initial aspects led physicians to erroneously propose this diagnosis: i) the initial traumatism a few weeks before the discovery of the tumour, ii) the first MRI observation which showed an extraosseous mass with a mature calcification peripherally predominant, iii) and the pathological aspects of mature lamellar bone synthesis by non atypical cells without abnormal mitoses. However, the radiological modifications observed later with the development of a centrifuge ossification corresponding to microscopic features to a reverse pattern of ossification ("reverse zonal phenomenon"), and of course, the progressive tumour growth and extension were that of a malignant tumour. Due to the initial misdiagnosis followed by the absence of follow-up, and the patient's refusal of surgery, the correct diagnosis was done too late and evolution was fatal.

Today, molecular analyses may resolve the diagnostic dilemma between low-grade ESOS and benign lesions. Amplification of genes in the 12q13-15 region, such as *SAS*, *CDK4 *and *MDM2*, is relatively frequent in osteosarcoma, notably in low-grade parosteal OS, making them suitable as markers for distinguishing them from benign ossifying [6-9]. In our patient, IHC and FISH analyses for *MDM2 *and *CDK4 *were done on 2006 from paraffin-embedded tumour samples biopsied on 2004. They showed the overexpression and amplification of these two oncogenes, thus ruling out the diagnosis of myositis ossificans and confirming that of sarcoma. To our knowledge, it is the first case of low-grade ESOS with documented molecular alterations.

## Conclusion

Our case is the seventh case of low-grade ESOS reported in literature, and the first case located in the chest wall. It highlights the importance and difficulty of early diagnosis of this very rare tumour, which may be confused with numerous benign diseases, notably myositis ossificans. Misdiagnosis may be fatal, as surgery is the only curative approach for these patients. In this context, it is worth noting the importance of molecular analysis (amplification and/or ovexpression of *MDM2 *and *CDK4*) to help diagnosis in ambiguous cases.

## Consent

Written informed consent was obtained from the patient's relatives for publication of this case report and any accompanying images.

## Competing interests

The authors declare that they have no competing interests.

## Authors' contributions

RS and FB conceived and designed the study, and wrote the manuscript. FB, PJW, PV, AS and IBR participated in patient's management. CB, GdP, FP, BC made the pathological and molecular explorations. All authors read and approved the final manuscript.

## Pre-publication history

The pre-publication history for this paper can be accessed here:

http://www.biomedcentral.com/1471-2407/10/645/prepub
